# Postpartum glucose intolerance after gestational diabetes mellitus: tailored prediction according to data-driven clusters and BMI-categories

**DOI:** 10.3389/fendo.2024.1381058

**Published:** 2024-07-16

**Authors:** Anna Lesniara-Stachon, Emmanuel Cosson, Alain Lacroix, Sybille Schenk, Dan Yedu Quansah, Jardena J. Puder

**Affiliations:** ^1^ Obstetric Service, Department Woman-Mother-Child, Lausanne University Hospital, Lausanne, Switzerland; ^2^ Department of Endocrinology-Diabetology-Nutrition, AP-HP, Avicenne Hospital, Paris 13 University, Sorbonne Paris Cité, CRNH-IdF, CINFO, Bobigny, France; ^3^ Sorbonne Paris Cité, UMR U1153 Inserm/U1125 Inra/Cnam/Université Paris 13, Bobigny, France; ^4^ Institute of Higher Education and Research in Healthcare (IUFRS), University of Lausanne, Lausanne, Switzerland; ^5^ Service of Endocrinology, Diabetes and Metabolism, Lausanne University Hospital, Lausanne, Switzerland

**Keywords:** clusters, insulin resistant, insulin deficient, gestational diabetes, glucose intolerance, predictors

## Abstract

**Objectives:**

To account for the heterogeneity of gestational diabetes (GDM), this study investigated tailored predictors during pregnancy and at 6-8 weeks postpartum of glucose intolerance (GI) at 1-year postpartum. We identified predictors according to data-driven clusters, analogous to the newly proposed diabetes classification, and for clinical ease also based on BMI-categories.

**Methods:**

This is a secondary analysis of the MySweetheart trial. It included 179 women with GDM who underwent a 75g oral glucose tolerance test and HbA1c measurement at 1-year postpartum. Predictors were determined according to: a) cluster analysis based on age, BMI, HOMA-IR and HOMA-B; and b) BMI-categories (normal weight [NW], and overweight/obesity [OW/OB]).

**Results:**

We identified two clusters during pregnancy and at 6-8 weeks postpartum (for both time points an “insulin-resistant”, and an “insulin-deficient” cluster). The “insulin-resistant” cluster was associated with a 2.9-fold (CI: 1.46-5.87; pregnancy) and 3.5-fold (CI: 1.63-7.52; at 6-8 weeks postpartum) increased risk of GI at 1-year postpartum. During pregnancy, the most relevant predictors of GI were history of previous GDM and fasting glucose for the “insulin-deficient” and NW category and HOMA-IR for the “insulin-resistant” and OW/OB category (all p ≤0.035). In the postpartum, predictors were more heterogenous and included the insulin-sensitivity-adjusted-secretion index and 1-h glucose in the “insulin-deficient” and NW women.

**Main conclusions:**

In women with GDM, we identified “insulin-resistant” and “insulin-deficient” clusters with distinct risks of future GI. Predictors varied according to clusters or BMI-categories emphasizing the need for tailored risk assessments.

## 1 Introduction

Women with gestational diabetes mellitus (GDM) have a 7-10-fold higher risk of incident diabetes (1) and an increased risk for future cardiovascular disease (2, 3). However, the population of GDM, just as observed in the population of subjects with diabetes, is not homogenous (4, 5). Early identification of women with GDM at the highest risk of future diabetes is crucial for prevention and long-term follow up (6).

Diabetes heterogeneity has recently been illustrated by a new classification system based on six variables (age, BMI, HbA1c, an estimate of the homoeostasis model assessment of β-cell function (HOMA-B), insulin resistance (HOMA-IR) and the presence or absence of islet autoantibodies) with distinct risks for complications (7, 8). The potential clustering of these variables and its impact on future diabetes risk has not been investigated in women with GDM.

Although GDM is generally characterized by increased insulin resistance and reduced insulin secretion (9), considerable heterogeneity exists (4, 5). Compared to women with normal glucose tolerance, half of women with GDM have increased insulin resistance and a third have reduced insulin secretion (4). While a previous study found that women with reduced insulin secretion did not differ from their normal glucose tolerant counterparts regarding BMI, fasting glucose in pregnancy, and the risk of adverse obstetric and neonatal outcomes, those with higher insulin resistance had a higher BMI, fasting glucose, and a higher risk of adverse pregnancy outcomes (4, 10).

Higher maternal age, pre-pregnancy weight and BMI, early diagnosis of GDM, the need for insulin treatment during pregnancy, glucose level and HbA1c in the third trimester have been identified as predictors of future diabetes or prediabetes in women with GDM (11–13). Other predictors of future glucose intolerance (GI) include a family history of type 2 diabetes, high-risk ethnicity, and previous GDM (14, 15). Although eating behaviour (intuitive eating) or dietary intake such as low-fat and low protein diet can predict reduced future diabetes risk after GDM (16, 17), the independent role of nutritional intake or eating behavior among women with GDM during pregnancy on future glucose intolerance or diabetes has not been previously investigated. Only few studies have investigated predictors of GI in the postpartum and this has been shown mainly in Asian populations (18). Previous studies have focused on predictors for all women together. However, predictors of GI might vary according to GDM subtypes (4, 10, 19). To account for the heterogeneity of GDM, more precise insight into different predictors according to GDM clusters or BMI-categories can help to better understand different risk factors within each subgroup.

We studied an exhaustive list of predictors (both in pregnancy and in the early postpartum) of GI in all women with GDM together and in GDM subgroups. Subgroups were investigated according to (a) a data-driven cluster analysis based on age, BMI, HOMA-IR and HOMA-B indices (8); and for clinical ease according to (b) normal weight (NW) vs overweight or obesity (OW-OB). This was done to identify the characteristics of the clusters and their metabolic risk profile and to propose a tailored approach for screening and treatment.

## 2 Materials and methods

### 2.1 Study design and participants

This study is a secondary analysis of the MySweetheart Trial (*NCT02890693*) (20), which tested the effect of an interdisciplinary lifestyle and psychosocial intervention on metabolic and mental health outcomes in women with GDM. Details of the MySweetheart trial have been already described (20). In total, we included 211 (105 in the intervention, 106 women in the usual care group) pregnant women, aged ≥18 years, with GDM diagnosed between 24-32 weeks of gestation by a 2-h 75g oral glucose tolerance test (oGTT), based on the IADPSG criteria (21, 22). Among them, 179 women completed the 1-year postpartum visit and were included in this analysis (20). The reasons for follow-up loss are shown in **Supplementary Figure 2.**

#### 2.1.1 Usual care group

The active lifestyle and guidelines-based usual care group received standard perinatal care according to the guidelines of the ADA and of the Endocrine Society (21, 23). Initial treatment was focused on nutritional therapy, increased physical activity and on gestational weight gain (GWG) recommendations according to the Institute of Medicine guidelines (24). After GDM diagnosis, women were seen at 24-32 weeks of gestation by a physician, or by a diabetes-specialist nurse and were followed-up until delivery [see details (20)]. They also had one appointment with a dietician for individualized dietary advice.

#### 2.1.2 Intervention group

On top of the usual care, the intervention program had four clinical lifestyle visits in pregnancy and in the postpartum. Women were invited to participate in group workshops, one in the pregnancy and one in the postpartum, and were followed by a lifestyle coach mostly through phone calls. The intervention focused on improving eating regulation, diet quality, increasing physical activity, providing mental health and social support, improving adherence to GWG recommendations and weight maintenance (20).

At 6-8 weeks and 1-year postpartum, all women underwent a 75-g oGTT along with an HbA1c measurement and then had a visit with the physician or nurse along with the dietician to discuss the results and receive adapted weight management and lifestyle counseling. No medical treatment was introduced until the 1-year postpartum visit.

### 2.2 Primary outcome

The main outcome of this study was GI (both prediabetes and diabetes) at 1-year postpartum according to ADA criteria (21). Prediabetes was diagnosed if fasting blood glucose was 5.6-6.9 mmol/l and/or 2-h blood glucose was 7.8-11 mmol/l, following a 75 g oGTT and/or HbA1c level 5.7- 6.4%. Diabetes was diagnosed if fasting blood glucose was ≥ 7 mmol/l and/or 2-h blood glucose was ≥ 11.1 mmol/l, following a 75 g oGTT and/or HbA1c level ≥ 6.5%.

### 2.3 Predictors of GI

Predictor variables were assessed at the first GDM visit (24-32 weeks of gestation) and/or at 6-8 weeks postpartum and included a personal medical history, socio-demographic, clinical, and laboratory measures, as well as eating behavior and nutritional intake (only in pregnancy), and detailed measures of insulin resistance and secretion (6-8 weeks postpartum).

#### 2.3.1 General predictors

Information on socio-demographic characteristics was collected at the first GDM visit and used as predictors both in pregnancy and at 6-8 weeks postpartum. This included age, education level and ethnicity. Data on medical characteristics were extracted from the women’s medical charts and included: family history of diabetes (first degree, second degree, no), previous history of GDM (yes/no), smoking during pregnancy (yes, no, stopped since knowledge of pregnancy), gravida, parity and medical treatment during pregnancy (no, metformin, insulin, insulin and metformin).

#### 2.3.2 Predictors assessed only in pregnancy

##### 2.3.2.1 Dietary intake assessment

We assessed dietary intake at the first GDM visit using a validated food-frequency questionnaire (FFQ) (25). We calculated daily total carbohydrate, protein, and fat intake (in gr), intake of monosaccharides, polysaccharides, animal protein, plant protein, cholesterol (mg), monounsaturated fat, polyunsaturated fat, total fiber (all in gr) and total energy intake (in kcals) (intake of alcohol was excluded), according to the French food composition table CIQUAL.

##### 2.3.2.2 Intuitive eating behavior

Intuitive eating (IE) was assessed at the first GDM visit with a 14-item self-report questionnaire consisting of two subscales of the French-adapted version of Intuitive Eating Scale-2 (IES-2) the “Eating for physical rather than emotional reasons” (EPR, 8 items) and the “Reliance on hunger and satiety cues” (RHSC, 6 items) (20, 26). A higher EPR subscale score reflects eating as a response to hunger and a lower score reflects eating to cope with emotional distress, while a higher RHSC subscale score indicates trust in internal cues and a lower score indicates less ability to regulate food intake.

#### 2.3.3 Predictors assessed both in pregnancy and at 6-8 weeks postpartum

##### 2.3.3.1 Clinical measures including anthropometry, body composition and blood pressure

Height was measured at the first GDM visit (to the nearest 0.1 cm), and weight was measured at all visits to the nearest 0.1 kg. Pre-pregnancy weight was taken from the participants’ medical charts or was self-reported when not available. Pre-pregnancy BMI and BMI at 6-8 weeks postpartum (kg/m^2^) were calculated. GWG was defined as the difference between pre-pregnancy weight and weight at the end of pregnancy. Total fat mass was measured using Bioelectrical Impedance Analysis (BIA) (Akern BIA 101), validated in pregnancy, at both visits and estimated from reactance and resistance values according to Kyle equation (27). Blood pressure was measured at both visits using a clinically validated sphygmomanometer (OMRON HEM-907, Japan).

##### 2.3.3.2 Laboratory measures, insulin sensitivity and resistance indices

Fasting glucose, insulin, lipids levels (HDL, LDL, cholesterol, triglycerides) were measured in pregnancy and at 6-8 weeks postpartum. We calculated Homeostatic Model Assessment for Insulin Resistance (HOMA-IR) and Homeostasis Model Assessment of β-cell function/insulin secretion (HOMA-B) (28). HbA1c was measured, by Afinion^®^ during pregnancy, and by high-performance liquid chromatography at 6-8 weeks postpartum.

#### 2.3.4 Predictors assessed only at 6-8 weeks postpartum

Information about breastfeeding (yes, no, duration) was obtained during the postpartum visit. Weight retention at 6-8 weeks postpartum was calculated as difference in weight between weight at 6-8 weeks postpartum and pre-pregnancy weight.

At 6-8 weeks postpartum, we performed an oGTT with glucose and insulin sampling at 30 min intervals for 2-h and we investigated 1-h and 2-h glucose values as predictors (29). We calculated the MATSUDA index (total body insulin sensitivity), insulin secretion (Area under the curve [AUC] and Insulinogenic Index [IGI]). We also determined the insulin sensitivity-adjusted secretion index or the insulin disposition index (ISSI-2) that adjusts insulin secretion for ambient insulin sensitivity (30, 31).

### 2.4 Statistical analyses

Data were analyzed using STATA version 15.1 (StataCorp LLC, TX, USA, 2017). Demographic and other descriptive variables were presented as means and standard deviations or percentages where appropriate (**Table 1**; **Supplementary Tables 1.A, 1.B**). Outcome data were normally distributed.

**Table 1:** Descriptive characteristics of the 179 study participants according to pre-pregnancy BMI-categories.

Variable	All women	NW^*^	OW/OB^*^
**Age (year)**	33.6 ± 4.9	33.8 ± 4.5	33.4 ± 5.4
**Gestational age at the first GDM visit**	30.7 ± 1.9	31.1 ± 1.8	30.3 ± 2.0
Educational level			
Obligatory education uncompleted	2 (1.3%)	0	1 (1.4%)
Obligatory education completed	21 (13.9%)	7 (8.3%)	14 (19.7%)
Upper secondary school	16 (10.6%)	9 (10.7%)	7 (9.9%)
General and professional formation	32 (21.2%)	11 (13.1%)	21 (29.6%)
Higher formation	80 (53.0%)	52 (61.9%)	28 (39.4%)
Ethnic origin			
Switzerland	52 (32.1%)	25 (29.1%)	27 (36.0%)
Europe East	14 (8.6%)	11 (12.8%)	3 (4.0%)
Europe West	53 (32.7%)	28 (32.6%)	25 (33.3%)
Asia	13 (8.0%)	7 (8.1%)	5 (6.7%)
Africa	21 (13.0%)	10 (11.6%)	11 (14.7%)
Latin America	7 (4.3%)	4 (4.7%)	3 (4.0%)
North of America	2 (1.2%)	1 (1.2%)	1 (1.3%)
Family history of Diabetes Mellitus			
1st degree	59 (34.1%)	30 (33.0%)	29 (34.1%)
2nd degree	51 (31.3%)	24 (26.1%)	26 (30.6%)
No	67 (34.6%)	37 (40.7%)	30 (35.3%)
History of GDM			
Yes	19 (23.8%)	8 (79.0%)	11 (23.4%)
No	67 (76.2%)	30 (21.0%)	36 (76.6%)
Smoking status during pregnancy			
Yes	22 (12.5%)	11 (11.7%)	10 (12.0%)
No	152 (86.4%)	81 (86.2%)	71 (85.5%)
Stopped since knowledge of pregnancy	2 (1.1%)	0 (0%)	2 (2.5%)
Gravida			
1	78 (43.6%)	47 (50.0%)	31 (36.5%)
2	41 (22.9%)	20 (21.3%)	21 (24.7%)
≥3	60 (33.5%)	27 (28.7%)	33 (38.8%)
Parity			
0	102 (57.0%)	61 (64.9%)	42 (49.4%)
1	49 (27.4%)	20 (21.3%)	29 (34.1%)
≥2	28 (15.6%)	13 (13.8%)	14 (16.5%)
Glucose-lowering medical treatment during pregnancy			
None	92 (53.5%)	56 (63.6%)	35 (42.2%)
Metformin	8 (4.7%)	2 (2.02%)	6 (7.2%)
Insulin	70 (40.7%)	30 (34.1%)	40 (48.2%)
Insulin and metformin	2 (1.2%)	0	2 (2.41%)
**Weight before pregnancy (kg)**	69.0 ± 14.9	58.5 ± 6.8	80.9 ± 12.3
**Pre-pregnancy BMI (kg/m^2^)**	25.6 ± 5.2	21.8 ± 2.1	29.8 ± 4.2
**Weight at the 1^st^ GDM visit (kg)**	79.2 ± 14.5	70.0 ± 8.9	89.9 ± 12.3
**Weight at 6-8 weeks postpartum (kg)**	73.2 ± 14.6	63.6 ± 8.5	83.7 ± 12.7
Diagnosis at 6-8 weeks postpartum ^a^			
Normal glucose tolerance	128 (71.1%)	68 (72.34%)	60 (70.6%)
Glucose intolerance (diabetes and prediabetes)	51 (28.9%)	26 (27.7%)	25 (29.4%)
**Weight at 1-year postpartum (kg)**	72.2 ± 16.1	61.6 ± 9.1	83.9 ± 13.8
Diagnosis at 1-year postpartum^b^			
Normal glucose tolerance	112 (62.6%)	70 (74.5%)	42 (49.4%)
Glucose intolerance (diabetes and prediabetes)	67 (37.4%)	24 (25.5%)	43 (50.6%)

Results are expressed as mean (± SD) for continuous variables and as number of participants (and their percentage) for categorical variables.

^*^Based on pre-pregnancy BMI: women with normal weight (NW) vs with pre-pregnancy overweight/obesity (OW/OB).

a NW: n= 28 with GI (n=25 prediabetes, n=3 diabetes), OW/OB: n=31 with GI (n=28 prediabetes, n=3 diabetes).

b NW: n= 24 with GI (n=21 prediabetes, n=3 diabetes), OW/OB: n=43 with GI (n=36 prediabetes, n=7 diabetes).

We adapted the data-driven cluster analysis mentioned in the introduction (7, 8). We kept four of their six variables used (pre-pregnancy age, BMI, HOMA-B and HOMA-IR) and did not use HbA1c and presence or absence of islet autoantibodies, as the ranges for HbA1c during pregnancy are much smaller, and the prevalence of autoantibodies in pregnancy is less than 10% (32, 33). In pregnancy, we used pre-pregnancy BMI and in the early postpartum we used BMI at 6-8 weeks postpartum. HOMA-B and HOMA-IR were used at the respective time point (pregnancy or early postpartum; **Supplementary Table 2**). As we did not yet know the output we would get, we opted for an unsupervised approach. We performed an unlabeled cluster analysis and used the Elbow and Silhouette methods to estimate the optimal numbers of clusters and a k-means clustering algorithm to classify the patients. Outliers were identified using a Mahalanobis algorithm on the variables used for the cluster selection and removed from the cluster analysis. These outliers were later on reclassified as “insulin-resistant” based on their values of the variables used for the clustering analysis.

As in clinical practice, it is difficult to assign patients according to the more complex clusters, we also performed stratified analyses for women according to BMI-categories. In pregnancy, women were stratified according to pre-pregnancy BMI and in the early postpartum according to BMI-categories at 6-8 weeks postpartum [‘normal weight’ [NW] with BMI: ≤24.9 kg/m^2^ and ‘overweight or obese’ [OW/OB] with BMI: ≥25 kg/m^2^]. For clinical reasons, we chose NW vs OW/OB instead of the exact median, as 52.5% of women were NW before pregnancy. In supplementary analyses, we also looked at the high vs low HOMA-IR and high vs low HOMA-B subgroups.

We determined the distribution of patients in the respective clusters and BMI-categories, and concordance between clusters in pregnancy and in the postpartum using Chi-square tests. We estimated the prevalence of GI at 1-year postpartum in the clusters or according to BMI-categories using Chi-square test and logistic regression analyses to assess the risk of GI.

We performed stratified analyses to investigate different predictors of GI at 1-year postpartum for women within clusters and BMI-categories both in pregnancy and in the early postpartum. To identify the best predictors for GI at 1-year postpartum, predictors that were significant in the univariate analyses (p<0.05) were entered in a stepwise multiple logistic regression model and are named in the respective legends of **Tables 2**, **3** and **Supplementary Tables 3.A, 3.B**, **4.A, 4.B**. Tested predictors included medical history, anthropometric, clinical, laboratory measures, several insulin resistance and secretion indices (see section above for details).

**Table 2:** Tailored predictors during pregnancy of GI at 1-year postpartum for all GDM women and according to clusters and BMI-categories.

	Predictors	N	OR	95% C.I.		P value
				Lower	Upper	
All women^a^	History of GDM	122	4.91	1.13	21.32	0.034
	Fasting glucose	122	3.39	1.25	9.23	0.017
	Diastolic blood pressure	122	1.07	1.01	1.12	0.013
	Fasting insulin	122	1.07	1.01	1.13	0.031
Insulin-deficient cluster^b^	History of GDM	88	12.28	2.04	74	0.006
	Fasting glucose	88	4.78	1.12	20.41	0.035
**Insulin-resistant cluster** ^c^	HOMA-IR	55	1.92	1.17	3.15	0.01
NW* ^d^	History of GDM	70	8.67	1.21	61.94	0.031
	Fasting glucose	70	7.82	1.64	37.28	0.01
OW/OB* ^e^	HOMA-IR	75	1.37	1.05	1.78	0.019

^*^NW, Women with pre-pregnancy normal weight; OW/OB, Women with pre-pregnancy overweight/obesity.

HOMA-IR, Homeostatic Model Assessment for Insulin Resistance.

a Predictors entered in the multivariate analysis: first pregnancy, previous history of GDM, pre-pregnancy BMI, BMI, weight, fasting glucose, HbA1c, fasting insulin, HOMA-IR, fat mass, systolic and diastolic blood pressure, glucose lowering treatment in pregnancy.

b Predictors entered in the multivariate analysis: first pregnancy, previous history of GDM, pre-pregnancy BMI, weight, fasting glucose in pregnancy.

c Predictors entered in the multivariate analysis: HbA1c, fasting insulin, HOMA-IR in pregnancy.

d Predictors entered in the multivariate analysis: previous history of GDM, pre-pregnancy BMI, HOMA-IR, fasting glucose in pregnancy.

e Predictors entered in the multivariate analysis: HbA1c, pre-pregnancy BMI, HOMA-IR, fasting insulin in pregnancy.

**Table 3:** Tailored predictors at 6-8 weeks postpartum of GI at 1-year postpartum for all GDM women and according to clusters and BMI-categories.

	Predictors	N	OR	95% C.I.		P value
				Lower	Upper	
All women^a^	1-h glucose	99	1.51	1.14	2.0	0.004
	Pre-pregnancy BMI	99	1.19	1.07	1.31	0.001
**Insulin-deficient cluster** ^b^	ISSI-2	80	0.26	0.1	0.64	0.004
**Insulin- resistant cluster** ^c^	HbA1c	39	6.56	1.09	39.32	0.04
NW* ^d^	1-h glucose	47	1.58	1.08	2.3	0.019
OW/OB* ^e^	GWG	89	0.98	-0.97	-1.03	0.017
	BMI	89	1.02	1.00	1.05	0.034
	2-h glucose	89	1.09	1.02	1.18	0.016
	Diastolic blood pressure	89	1.02	1.01	1.03	0.002
	History of GDM	89	1.31	1.01	1.69	0.042

*NW, Women with normal weight at 6-8 weeks postpartum; *OW/OB, Women with overweight/obesity at 6-8 weeks postpartum.

ISSI-2, insulin sensitivity-adjusted secretion index; HbA1c, Glycated hemoglobin; 1-h glucose, glucose 1-h during oGTT; 2-h glucose, glucose 2-h during oGTT.

a Predictors entered in the multivariate analysis: previous history of GDM, first pregnancy, glucose lowering treatment in pregnancy, pre-pregnancy BMI, weight, triglycerides, BMI, fat mass, systolic and diastolic blood pressure, fasting glucose, 1-h glucose, 2-h glucose, fasting insulin, HOMA-IR, Matsuda index., HbA1c, IGI, ISSI-2, HOMA-B at 6-8 weeks postpartum.

b Predictors entered in the multivariate analysis: pre-pregnancy BMI, glucose lowering treatment in pregnancy, weight, BMI, systolic blood pressure, 1-h glucose, 2-h glucose, ISSI-2 at 6-8 weeks postpartum.

c Predictors entered in the multivariate analysis: first pregnancy, HbA1c at 6-8 weeks postpartum.

d Predictors entered in the multivariate analysis:1-h glucose, 2-h glucose, ISSI-2 at 6-8 weeks postpartum.

e Predictors entered in the multivariate analysis: previous history of GDM, pre-pregnancy BMI, gestational weight gain, weight, BMI, fat mass, systolic and diastolic blood pressure, fasting glucose, Matsuda index, 1-h glucose, 2-h glucose, ISSI-2 at 6-8 weeks postpartum.

As previous history of GDM necessitates a previous pregnancy to have a precise documentation and 43,6% of the women were pregnant for the first time, we also performed predictors analyses in pregnancy without the variable “previous history of GDM” (**Supplementary Table 4.A**). As ISSI-2 is more complex measure, we also performed predictors analysis without this variable (**Supplementary Table 4.B**).

In all analyses, predictors, outcomes, and effect sizes were similar for the intervention and the active usual care group. Thus, women from both groups were pooled together and all analyses were adjusted for group allocation. In the different final models, we did not observe any interaction between the predictor variables and the group allocation. We did not perform any imputation due to loss of observations or missing data. Statistical significance was defined at the two-sided α level of <0.05.

## 3 Results

### 3.1 Baseline characteristics and changes between pregnancy/early postpartum and 1-year postpartum

**Table 1** shows the baseline characteristics of all 179 of the initial 211 women that completed the 1-year postpartum visit. For clinical ease, data are also shown according to pre-pregnancy BMI-categories. Within the OW/OB category, 42 (40,8%) women were obese. At 1-year postpartum, 32.22% (n=58) had prediabetes and 5.03% (n=9) diabetes (together n=67 with GI). There were no significant differences in age or pre-pregnancy weight between the 179 women who completed and the 32 women who did not complete the 1-year postpartum visit. The flow sheet and reasons for drop-out are shown in the **Supplementary Figure 2**. To describe the metabolic characteristics and dietary intakes of the population, **Supplementary Tables 1.A, 1.B** show their descriptive values at baseline, 6-8 weeks, and 1-year postpartum as well as their changes.

#### 3.1.1 Cluster characteristics and prediction of GI by cluster and BMI-categories

**Figure 1:**
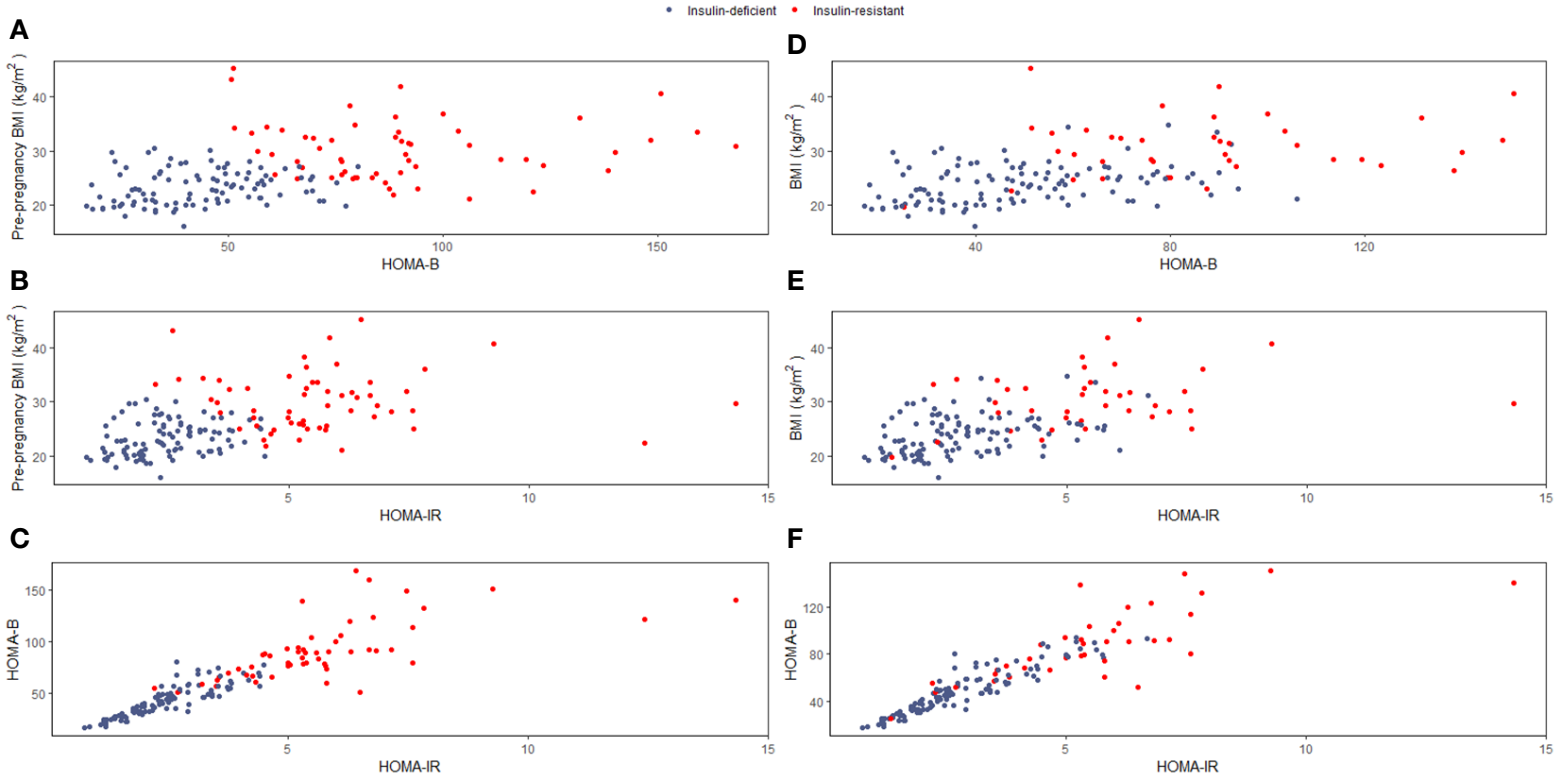
**(A–F)**. Cluster characteristics We used the Elbow and Silhouette methods to estimate the optimal numbers of clusters and a k-means clustering algorithm to classify the patients in pregnancy **(A–C)** and at 6-8 weeks postpartum **(D–F)**. **(A)** pre-pregnancy BMI and HOMA-B in pregnancy, **(B)** pre-pregnancy BMI and HOMA-IR in pregnancy, **(C)** HOMA-B and HOMA-IR in pregnancy, **(D)** BMI and HOMA-B at 6-8 weeks postpartum, **(E)** BMI and HOMA-IR at 6-8 weeks postpartum, **(F)** HOMA-B and HOMA-IR at 6-8 weeks postpartum BMI, Body Mass Index; HOMA-B, homoeostatic model assessment of β-cell function (insulin secretion); HOMA-IR, homoeostatic model assessment of insulin resistance.

We identified two clusters (“insulin-resistant” and “insulin-deficient”) in pregnancy and in the early postpartum (**Figure 1**; **Supplementary Figure 1**).

In pregnancy, the “insulin-resistant” cluster (55/154 women, 35.7%) was characterized by higher pre-pregnancy BMI, HOMA-IR and HOMA-B compared to the “insulin-deficient” cluster (99/154 women, 64.3%) (**Figures 1A–C**; **Supplementary Table 2**). The risk of GI at 1-year postpartum was 2.93 times (95% CI: 1.46-5.87, p=0.002) higher among women in the “insulin-resistant” cluster compared to the “insulin-deficient” one.

At 6-8 weeks postpartum, the “insulin-resistant” cluster (39/152, 25.7%) included women with higher BMI, HOMA-IR and HOMA-B compared to women in the “insulin-deficient” cluster (113/154, 74.3%) (**Figures 1D–F**; **Supplementary Table 2**). The risk of GI at 1-year postpartum was 3.50 times (95% CI: 1.63-7.52, P=0.001) higher among women in the “insulin-resistant” cluster compared to the “insulin-deficient”.

Tailored prediction of GI at 1-year postpartum was also performed according to BMI-categories. The risk of GI in women in the OW/OB in pregnancy was 2.99-fold (95% CI: 1.59-5.60, P=0.001) higher, and in the early postpartum 3.62-fold (95% CI: 1.75 -7.50, P=0.001) higher compared to the NW category.

There was a high concordance between the “insulin-deficient” cluster and the NW category: most women in the NW category also belonged to the “insulin-deficient” cluster (88.6% in pregnancy and 98.1% in the postpartum), while there was consistency for the “insulin-resistant” cluster and the OW/OB category (61.4% of OW/OB women in pregnancy and 39.6% in the postpartum also belonged to the “insulin-resistant” cluster). There was also a high agreement between the “insulin-deficient” clusters in pregnancy and in the postpartum (97.94%), while the concordance for the “insulin-resistant” clusters was 66.67%.

### 3.2 Tailored predictors of GI in each cluster and BMI-categories

#### 3.2.1 Predictors in pregnancy

Predictors that were significant in the univariate analyses are described in the legends of **Table 2** and of the **Supplementary Tables 3.A**, **4.A**. In a stepwise multivariable analyses, significant predictors for GI at 1-year postpartum for all women were fasting glucose, insulin, diastolic blood pressure in pregnancy and previous history of GDM (all p ≤ 0.034, **Table 2**). Predictors of GI at 1-year postpartum in the “insulin-deficient” cluster and in NW category were the same, i.e., a previous history of GDM and fasting glucose (all p ≤ 0.035). Similarly, in the “insulin-resistant” cluster, and in OW/OB category, the best predictor of GI was HOMA-IR (all p ≤ 0.019). None of the dietary intake variables or intuitive eating scores significantly predicted GI. Our supplementary analysis according to HOMA-IR/HOMA-B subgroups in pregnancy showed similar findings, i.e., a history of GDM/fasting glucose as significant predictors of GI at 1-year postpartum for low HOMA-IR/HOMA-B subgroups (all p ≤ 0.026) and HOMA-IR for high HOMA-IR/HOMA-B subgroups (all p ≤ 0.011) (**Supplementary Table 3.A**).

When analysing the predictors without “a previous history of GDM”, results were similar, i.e., fasting glucose, gravida and diastolic blood pressure in pregnancy were the best predictors for all women (all p ≤ 0.007), and fasting glucose for the “insulin-deficient” cluster and NW women (all p ≤ 0.004, **Supplementary Table 4.A**).

#### 3.2.2 Predictors in the early postpartum

Predictors that were significant in the univariate analyses are described in the legends of **Table 3** and of the **Supplementary Tables 3.B**, **4.B**. In stepwise multivariable analyses, predictors for GI at 1-year postpartum for all women were pre-pregnancy BMI and 1-h glucose during the oGTT (all p ≤ 0.004, **Table 3**). The latter was also the best predictor for GI in the NW category (p=0.019). In the “insulin-deficient” cluster, the most important predictor for GI at 1-year postpartum was ISSI-2 (all p ≤ 0.008). In the “insulin-resistant” cluster the best predictor for GI was HbA1c (p=0.04). In OW/OB women, predictors were heterogeneous and included GWG, BMI, 2-h glucose during the oGTT, diastolic blood pressure and history of GDM (all p ≤ 0.042). In supplementary analysis for HOMA-IR/HOMA-B subgroups, the most important predictor for GI at 1-year postpartum was ISSI-2 for low HOMA-IR and low HOMA-B subgroups (all p=0.008), and for high HOMA-IR and high HOMA-B it was pre-pregnancy BMI/1-h glucose (all p ≤ 0.044, **Supplementary Table 3.B**).

In the analysis without the ISSI-2, the most common predictor for all women and women with NW, OW/OB was the 1-h glucose during the oGTT (all p ≤ 0.038, **Supplementary Table 4.B**).

## 4 Discussion

This study used an adaption of the newly proposed diabetes-cluster classification to classify women with GDM based on age, BMI, HOMA-IR and HOMA-B. We compared this classification to a clinical classification according to BMI-categories. During pregnancy and in the postpartum, we identified both an “insulin-resistant” and an “insulin-deficient” cluster of women with GDM. The former had a 2.9-fold (for pregnancy) to 3.5-fold (for the early postpartum) increased risk of GI at 1-year postpartum. Tailored predictors in pregnancy for GI at 1-year postpartum were a history of GDM and fasting glucose for the “insulin-deficient” cluster, as well as for women in the NW BMI-category. For the “insulin-resistant” cluster or women with OW/OB, this was HOMA-IR. In the postpartum, we confirmed the utility of 1-h glucose during early postpartum as predictive for future GI especially when a more complex measure, ISSI-2 (or insulin sensitivity-adjusted insulin secretion), was removed from the analysis.

Overall, our data-driven analyses point towards the existence of two clusters with different risks of future GI in women with GDM. For clinical ease, BMI-category, particularly NW could represent a simple proxy for the “insulin-deficient” cluster, as we found a very high concordance between women with NW and the “insulin-deficient” cluster. In addition, belonging to the “insulin-resistant” cluster (as compared to the “insulin-deficient” one) increased the risk of GI at 1-year postpartum to a similar extent as belonging to the OW/OB category did when compared to NW women. Predictors of future GI also varied according to clusters and BMI-categories and were particularly specific and helpful in pregnancy, which points to the utility of a more personalized approach.

While Ahlqvist et al. classified patients with diabetes into five clusters with different characteristics and risks of diabetic complications outside pregnancy, our analyses in women with GDM identified two clusters. The characteristics of three of their clusters resemble the ones found in our population (severe insulin-deficient diabetes, mild obesity-related diabetes, severe insulin resistant diabetes). In analogy to Ahlqvist et al, glucose control also differed between our “insulin-resistant” cluster. Most women (98%) belonging to “insulin-deficient” cluster during pregnancy were also categorized as “insulin-deficient” in the postpartum. The concordance between the “insulin-resistant” clusters during pregnancy and the postpartum was 67%, i.e. a third of women in the “insulin-resistant” cluster during pregnancy “switched” the “insulin-deficient” cluster in the postpartum. This probably means that a third of women have a particularly pronounced increase in insulin resistance during pregnancy that is transient and reverses in the postpartum. In terms of complications, our “insulin-resistant” clusters had a 2.9-3.5 higher risk of later GI. Data-driven cluster analyses in this population may be useful for future metabolic risk assessment. For clinical feasibility, we also assessed the risk of GI in BMI-categories and could confirm similar results, i.e., we found higher risks in women with OW/OB, compared to NW women. This suggest that BMI, a simple and cheap measure, could serve as a proxy for part of the cluster analysis in this population.

Another study has used cluster analysis in pregnancy to assess the risk of offspring obesity in a general population of healthy pregnant women (34). Based on nine laboratory variables, they identified five clusters, among others an insulin resistant–hyperglycemic cluster that had the highest rates of obesity in the offspring. Previous studies in women with GDM did not perform cluster analysis, but performed subgroups, that were based on insulin resistance (Matsuda index or HOMA-IR) or on more complex insulin secretion measures (Stumvoll 1st phase index) (4, 10, 19, 35). They mainly investigated the relationship between GDM subgroups and pregnancy outcomes (10, 19, 36). Of those focusing on future risk of GI, two showed no differences between the subgroups (10, 35). We are only aware of one study that found differences in the risk of developing GI between subgroups of women with GDM. Their subgroups were based on family history of diabetes, the need for glucose-lowering treatment during pregnancy, and pre-pregnancy BMI (37). However, these studies (10, 35) did not examine tailored predictors of GI within these subgroups.

To further provide a more tailored insight and counseling, we assessed the predictors of GI according to clusters and BMI-categories. Most studies assessed predictors of GI for the entire population, grouping women with NW, OW and OB together (12, 38, 39). Similar to a few other studies (40, 41) we considered women with OW and OB separately from those with NW, but pooled them as one BMI category (OW/OB) and compared them to women with NW. Indeed, there is evidence of similar pattern of altered signaling pathways in the human foetoplacental microvascular and macrovascular endothelium in women with OW and OB, which contrasts with findings in NW women (42). In pregnancy, results showed that fasting glucose and a history of GDM were the most relevant predictors of GI for the “insulin-deficient” cluster, and in women with NW. For the “insulin-resistant” cluster and the OW/OB category it was insulin resistance (HOMA-IR). Thus, predictors of GI in pregnancy were similar within clusters or BMI-categories and could be attributed to the differences in insulin resistance or deficiency. This was additionally confirmed in supplementary analyses for low/high HOMA-IR and HOMA-B subgroups. We also showed that detailed evaluation of eating behavior or nutritional intake in pregnancy were not superior to simple measures. Previous studies have shown that higher vegetables or fruit intake as well as lower energy, protein, and animal fat intake in the postpartum was associated with lower risk of GI (43), but their results were not independent of other clinical and laboratory parameters like glucose control and insulin resistance/sensitivity indices.

In the early postpartum, ISSI-2 was the most important predictor of GI for the “insulin-deficient” cluster. Interestingly, the 1-h glucose level during the oGTT was an important predictor for all women together, and for the NW women. When removing the more complex ISSI-2 from the model, the 1-h glucose predicted GI in women with OW/OB. This is in line with findings of Göbl et al. (29), who showed that 1-h glucose levels during the oGTT in the postpartum were independently associated with a risk for overt diabetes among women with GDM up to 10-years of follow-up. Our results confirm the utility of the 1-h glucose value in the early postpartum as a predictor of future GI.

We propose a novel extension of the new diabetes-classification system to include women with GDM. Other strengths of this study are the use of tailored predictors for future GI based on BMI-categories and clusters, both in pregnancy and in the early postpartum. Compared to other studies, we used a very exhaustive list of clinical, laboratory and more complicated indices of insulin secretion/resistance as well as dietary intake and eating behaviour.

Limitations of our study include a relatively small sample size, particularly in some stratified subgroups, and the fact that we pooled women in the intervention and control group together. However outcomes and effect sizes were not different between groups, and we tested for interactions and always adjusted for group allocation. Additionally, we analyzed women with OW and OB as a single group, which might result in the loss of data. We did this to increase our sample size, as for some measures such as the FFQ or detailed measures of insulin resistance and sensitivity we would not have enough valid measures. A further limitation is the focus on pre-pregnancy BMI and not on body composition when categorizing women. However, we did not have any data about body composition before pregnancy. Furthermore, using body composition would be more cumbersome in a clinical setting. Of note, 80% of women in the OW/OB category based on pre-pregnancy BMI were also attributed to the category of high-fat (based on the fat mass in pregnancy assessed by bioimpedance: low, high fat mass using the median value as cut-off ≤ 30.87 kg) and 85% of women in the NW category based on pre-pregnancy BMI also to the low-fat category. Women were only followed-up until 1 year postpartum. Another limitation is the subjective assessment of dietary intake, using an FFQ. Moreover, several measures such as nutritional intake or the more complicated indices of insulin resistance and secretion were not assessed at all time points and there were some missing data, especially for more complicated indices. The unsupervised rather than supervised approach taken in this study for the clustering analysis was due to the fact that our data was unlabeled and we were interested to describe groups, patterns and relationships. With this approach, there is no use to train and validate our current model in a separate population, but another approach could be used in future studies. The choice of a k-means algorithm was mostly to align our approach with previous studies. We also used a k-medoids analysis and got similar patient assignments to clusters (results not reported in this study). As this cluster classification of women with GDM has been only shown in this study, a replication of our results would be needed to increase its generalizability and external validity.

Overall, we identified two clusters in women with GDM (an “insulin-resistant” and an “insulin-deficient” cluster) with distinct risks of future GI. Predictors varied according to clusters and BMI-categories emphasizing the need for tailored risk assessments.

## Group members of MySweetHeart research group

Amar Arhab, Pascal Bovet, Arnaud Chiolero, Stefano Di Bernardo, Adina Mihaela Epure, Sandrine Estoppey Younes, Leah Gilbert, Justine Gross, Antje Horsch, Stefano Lanzi, Seyda Mayerat, Yvan Mivelaz, Jardena J Puder, Dan Yedu Quansah, Jean-Benoit Rossel, Nicole Sekarski, Umberto Simeoni, Bobby Stuijfzand, Yvan Vial.

## Data availability statement

The raw data supporting the conclusions of this article will be made available by the authors, without undue reservation.

## Ethics statement

The studies involving humans were approved by La Commission cantonale d’éthique de la recherche sur l’être humain (CER-VD). The studies were conducted in accordance with the local legislation and institutional requirements. The participants provided their written informed consent to participate in this study.

## Author contributions

AL-S: Conceptualization, Data curation, Investigation, Methodology, Software, Visualization, Formal analysis, Writing – original draft. EC: Writing – review & editing. AL: Formal analysis, Writing – review & editing. SS: Writing – review & editing. DYQ: Conceptualization, Data curation, Methodology, Visualization, Supervision, Writing – review & editing. JJP: Conceptualization, Data curation, Methodology, Supervision, Funding acquisition, Project administration, Visualization, Writing – review & editing.
