# Supplementary material for: Postpartum glucose intolerance after gestational diabetes mellitus: tailored prediction according to data-driven clusters and BMI-categories
Source: Front Endocrinol (Lausanne). 2024 Jul 16;15:1381058. doi: 10.3389/fendo.2024.1381058 (PMC11286585; doi:10.3389/fendo.2024.1381058)
Supplement: Supplementary file 1 [file DataSheet_1.docx]

**Supplementary tables:**

**Table 1.A** Descriptive values and changes in metabolic health outcomes and dietary measures between the baseline visit in the GDM clinic in pregnancy and 1-year postpartum in all women.

|  | **Baseline GDM visit in pregnancy** | **1-year postpartum** | **Mean difference** | **P value** |
| --- | --- | --- | --- | --- |
|  | **Mean & SD** | **Mean & SD** | **Mean & SD** |  |
| **BMI (kg/m^2^)** | 29.49 ± 4.92 | 26.89 ± 5.66 | -2.60 ± 1.84 | **<0.001** |
| **Weight (kg)** | 79.38 ± 14.84 | 72.50 ± 16.64 | -6.88 ± 5.35 | **<0.001** |
| **Fat mass by BIA (kg)** | 31.56 ± 9.24 | 26.65 ± 10.64 | -4.91 ± 4.29 | **<0.001** |
| **HDL (mmol/l)** | 1.77 ± 0.38 | 1.39 ± 0.38 | -0.37 ± 0.37 | **<0.001** |
| **LDL (mmol/l)** | 3.16 ± 0.98 | 2.55 ± 0.68 | -0.61 ± 0.85 | **<0.001** |
| **Cholesterol (mmol/l)** | 5.98 ± 1.07 | 4.42 ± 0.76 | -1.56 ± 0.91 | **<0.001** |
| **Triglycerides (mmol/l)** | 2.37 ± 0.74 | 1.05 ± 0.51 | -1.33 ± 0.77 | **<0.001** |
| **Diastolic blood pressure (mmHg)** | 71.22 ± 9.17 | 71.29 ± 8.71 | 0.07 ± 9.57 | 0.896 |
| **Systolic blood pressure (mmHg)** | 111.09 ± 10.30 | 111.47 ± 10.30 | 0.38 ± 12 | 0.608 |
| **Fasting glucose (mmol/L)** | 4.96 ± 0.49 | 5.38 ± 0.62 | 0.42 ± 0.59 | **<0.001** |
| **Fasting insulin (μU/mL)** | 15.96 ± 8.10 | 13.25 ± 8.74 | -2.71 ± 1.78 | **<0.001** |
| ***Insulin resistance/secretion*** | | | | |
| **HOMA-IR** | 3.59 ± 2.05 | 3.24 ± 2.32 | -0.35 ± 1.51 | **0.008** |
| **HOMA-B** | 60.23 ± 30.64 | 45.33 ± 31.56 | -14.9 ± 20.24 | **<0.001** |
| ***Dietary measures*** | | | | |
| **IES-2 score 1 ^a^** | 3.85 ± 0.83 | 3.85 ± 0.87 | -0.0009 ± 0.73 | 0.076 |
| **IES-2 score 2 ^b^** | 3.53 ± 0.82 | 3.64 ± 0.82 | 0.12 ± 0.88 | 0.989 |
| **Total energy (kcals)** | 1453.59 ± 553.02 | 1459.72 ± 550.94 | 6.13 ± 715.13 | 0.954 |
| **Total carbohydrates (gr)** | 635.80 ± 284.05 | 620.44 ± 293.90 | -15.36 ± 384.88 | 0.679 |
| **Monosaccharides (gr)** | 317.14 ± 17.49 | 304.53 ± 19.65 | -12.6 ± 230.26 | 0.593 |
| **Polysaccharides (gr)** | 316.79 ± 177.39 | 314.37 ± 159.49 | -2.42 ± 211.55 | 0.881 |
| **Total protein (gr)** | 252.98 ± 109.47 | 252.80 ± 108.33 | -0.18 ± 124.11 | 0.988 |
| **Animal protein (gr)** | 184.87 ± 94.41 | 186.35 ± 95.07 | 1.49 ± 100.76 | 0.883 |
| **Plant protein (gr)** | 68.02 ± 30.40 | 66.40 ± 29.95 | -1.62 ± 39.97 | 0.685 |
| **Total fat (gr)** | 564.81 ± 249.07 | 586.48 ± 239.87 | 21.67 ± 279.02 | 0.474 |
| **Cholesterol (mg)** | 297.35 ± 159.90 | 323.70 ± 195.24 | 26.35 ± 182.83 | 0.179 |
| **Monounsaturated fat (MUFA) (gr)** | 249.32 ± 123.36 | 256.10 ± 107.76 | 6.78 ± 122.43 | 0.606 |
| **Polyunsaturated fat (PUFA) (gr)** | 76.58 ± 31.68 | 78.67 ± 34.68 | 2.09 ± 44.88 | 0.663 |
| **Total fibre (gr)** | 14.86 ± 7.15 | 13.01 ± 7.22 | -1.85 ± 9.57 | 0.071 |

**^a^** Eating for physical rather than emotional reasons

**^b^** Reliance on hunger and satiety cues

*HOMA-IR: Homeostatic Model Assessment for Insulin Resistance, HOMA-B: Homeostasis Model Assessment of β-cell function/insulin secretion*

**Table 1.B** Changes in metabolic health outcomes between 6-8 weeks and 1-year postpartum in all women.

|  | **6-8 weeks postpartum** | **1-year postpartum** | **Mean difference** | **P value** |
| --- | --- | --- | --- | --- |
|  | **Mean & SD** | **Mean & SD** | **Mean & SD** |  |
| **BMI (kg/m^2^)** | 27.17 ± 5.03 | 26.91 ± 5.67 | -0.27 ± 1.80 | 0.053 |
| **Weight (kg)** | 73.23 ± 14.56 | 72.47 ± 16.17 | -0.76 ± 4.90 | **0.043** |
| **Fat mass by BIA (kg)** | 27.99 ± 9.45 | 26.59 ± 10.64 | -1.39 ± 4.09 | **<0.001** |
| **Diastolic blood pressure (mmHg)** | 71.51 ± 9.78 | 71.16 ± 8.69 | -0.36 ± 10.77 | 0.665 |
| **Systolic blood pressure (mmHg)** | 109.80 ± 11.04 | 111.32 ± 10.34 | 1.51 ± 11.64 | 0.067 |
| **HDL (mmol/l)** | 1.56 ± 0.42 | 1.39 ± 0.38 | -0.17 ± 0.29 | **<0.001** |
| **LDL (mmol/l)** | 3.12 ± 0.70 | 2.54 ± 0.67 | -0.58 ± 0.53 | **<0.001** |
| **Cholesterol (mmol/l)** | 5.23 ± 0.80 | 4.41 ± 0.76 | -0.81 ± 0.59 | **<0.001** |
| **Triglycerides (mmol/l)** | 1.17 ± 0.61 | 1.03 ± 0.43 | -0.14 ± 0.48 | **<0.001** |
| **Fasting glucose (mmol/L)** | 5.08 ± 0.68 | 5.38 ± 0.62 | 0.30 ± 0.69 | **<0.001** |
| **1-h glucose (mmol/L)** | 6.96 ± 1.98 | 7.31 ± 2.39 | 0.34 ± 2.13 | 0.05 |
| **2-h glucose (mmol/L)** | 5.36 ± 1.39 | 5.94 ± 1.70 | 0.58 ± 1.57 | **<0.001** |
| **Fasting insulin (μU/mL)** | 9.03 ± 7.52 | 13.02 ± 8.54 | 3.99 ± 6.29 | **<0.001** |
| ***Insulin resistance indices*** | | | | |
| **HOMA-IR** | 2.12 ± 1.90 | 3.21 ± 2.34 | 1.09 ± 1.67 | **<0.001** |
| **MATSUDA index** | 7.29 ± 3.92 | 5.02 ± 2.95 | -2.27 ± 2.7 | **<0.001** |
| ***Insulin secretion calculations*** | | | | |
| **AUC all** | 0.44 ± 0.21 | 0.52 ± 0.25 | 0.08 ± 0.17 | **0.001** |
| **IGI** | 129.23 ± 372.26 | 135.08 ± 229.34 | 5.85 ± 426.75 | 0.825 |
| **HOMA-B** | 31.18 ± 27.52 | 43.9 ± 29.05 | 12.712 ± 22.52 | **<0.001** |
| ***Disposition index*** | | | | |
| **ISSI-2** | 2.65 ± 1.02 | 2.14 ± 0.86 | -0.51 ± 0.91 | **<0.001** |

*HOMA-IR: Homeostatic Model Assessment for Insulin Resistance, AUC: area under curve, IGI: insulinogenic index, HOMA-B: Homeostasis Model Assessment of β-cell function/insulin secretion, ISSI-2: insulin sensitivity-adjusted secretion index or the insulin disposition index*

**Table 2.** Metabolic characteristics of the two clusters.

|  | **"Insulin-resistant” cluster** | **"Insulin-deficient” cluster** | **P-value** |
| --- | --- | --- | --- |
| **Clusters in pregnancy** | | | |
| **N (%)** | 55 (35.7%) | 99 (64.3%) |  |
| **Age (years)** | 31.07 ± 6.53 | 34.85 ± 4.48 | < 0.001 |
| **Pre-pregnancy BMI (kg/m^2^)** | 30.14 ± 5.36 | 23.25 ± 3.17 | < 0.001 |
| **HOMA-IR in pregnancy** | 5.61 ± 2.04 | 2.46 ± 0.89 | < 0.001 |
| **HOMA-B in pregnancy** | 90.04 ± 27.8 | 43.59 ± 15.33 | < 0.001 |
| **Fasting glucose at 1-year postpartum (mmol/L)** | 5.58 ± 0.73 | 5.24 ± 0.52 | 0.001 |
| **2-h glucose at 1-year postpartum (mmol/L)** | 6.23 ± 1.58 | 5.74 ± 1.72 | 0.072 |
| **HbA1c at 1-year postpartum (mmol/mol)** | 35.48 ± 7.11 | 33.96 ± 6.59 | 0.186 |
| **HbA1c at 1-year postpartum (%)** | 5.40 ± 0.65 | 5.26 ± 0.6 | 0.186 |
| **Presence of glucose intolerance at 1-year postpartum, n (%)** | 29/55 (52.73%) | 28/99 (28.28%) | 0.002 |
| **Clusters at 6-8 weeks postpartum** | | | |
| **N (%)** | 39 (25.7 %) | 113 (74.3%) |  |
| **Age (years)** | 30.1 ± 6.74 | 34.75 ± 4.7 | < 0.001 |
| **BMI at 6-8 weeks postpartum (kg/m^2^)** | 32.9 ± 5.24 | 25.26 ± 3.68 | < 0.001 |
| **HOMA-IR at 6-8 weeks postpartum** | 5.51 ± 2.23 | 2.83 ± 1.29 | < 0.001 |
| **HOMA-B at 6-8 weeks postpartum** | 87.05 ± 29.62 | 49.04 ± 20.34 | < 0.001 |
| **Fasting glucose at 1-year postpartum (mmol/L)** | 5.73 ± 0.79 | 5.25 ± 0.51 | < 0.001 |
| **2-h glucose at 1-year postpartum (mmol/L)** | 6.31 ± 1.64 | 5.79 ± 1.68 | 0.119 |
| **HbA1c at 1-year postpartum (mmol/mol)** | 34.79 ± 5.26 | 34.1 ± 6.36 | 0.548 |
| **HbA1c at 1-year postpartum (%)** | 5.33 ± 0.48 | 5.27 ± 0.58 | 0.548 |
| **Presence of glucose intolerance at 1-year postpartum, n (%)** | 23/39 (58.97%) | 34/113 (30.09%) | 0.001 |

*HOMA-IR: Homeostatic Model Assessment for Insulin Resistance, HOMA-B: Homeostasis Model Assessment of β-cell function/insulin secretion, HbA1c:* *Glycated hemoglobin*

**Table 3.A** Tailored predictors during pregnancy of glucose intolerance at 1-year postpartum according to HOMA-IR/HOMA-B subgroups.

|  | **Predictors** | **N** | **OR** | **95% C.I.** | | **P value** |
| --- | --- | --- | --- | --- | --- | --- |
|  |  |  |  | **Lower** | **Upper** |  |
| **Low HOMA-IR** ^a^ | History of GDM | 67 | 15.60 | 1.40 | 174.07 | 0.026 |
| **High HOMA-IR ^b^** | HOMA-IR | 76 | 1.62 | 1.12 | 2.36 | 0.011 |
| **Low HOMA-B ^c^** | History of GDM | 66 | 74.85 | 3.90 | 1434.96 | 0.004 |
|  | Fasting glucose | 66 | 47.37 | 4.40 | 509.73 | 0.001 |
| **High HOMA-B ^d^** | HOMA-IR | 74 | 1.87 | 1.22 | 2.87 | 0.004 |
|  | Diastolic blood pressure | 74 | 1.08 | 1.01 | 1.15 | 0.024 |
|  | HbA1c | 74 | 10.00 | 1.45 | 68.63 | 0.019 |

*HOMA-IR: Homeostatic Model Assessment for Insulin Resistance, HOMA-B: Homeostasis Model Assessment of β-cell function/insulin secretion, HbA1c:* *Glycated hemoglobin*

*HOMA-IR and HOMA-B were stratified according to their median values in pregnancy (HOMA-IR ≥ and < 3.128, HOMA-B ≥ and < 54.76)*

^a^ *Predictors entered in the multivariate analysis: previous history of GDM ,weight, fasting glucose in pregnancy*

^b^ *Predictors entered in the multivariate analysis: pre-pregnancy BMI, fasting insulin, HbA1c, HOMA-IR in pregnancy*

^c^ *Predictors entered in the multivariate analysis: first pregnancy, previous history of GDM, fasting glucose, HOMA-IR, weight in pregnancy*

^d^ *Predictors entered in the multivariate analysis: pre-pregnancy BMI, fasting glucose, fasting insulin, HOMA-IR, HOMA-B, weight, BMI, HbA1c, diastolic blood pressure in pregnancy*

**Table 3.B** Tailored predictors at 6-8 weeks postpartum of glucose intolerance at 1-year postpartum according to HOMA-IR/HOMA-B subgroups.

|  | **Predictors** | **N** | **OR** | **95% C.I.** | | **P value** |
| --- | --- | --- | --- | --- | --- | --- |
|  |  |  |  | **Lower** | **Upper** |  |
|  |  |  |  |  |  |  |
| **Low HOMA-IR ^a^** | ISSI-2 | 58 | 0.32 | 0.14 | 0.74 | 0.008 |
| **High HOMA-IR ^b^** | Pre-pregnancy BMI | 44 | 1.17 | 1.02 | 1.34 | 0.024 |
| **Low HOMA-B ^c^** | ISSI-2 | 58 | 0.32 | 0.14 | 0.74 | 0.008 |
| **High HOMA-B ^d^** | Pre-pregnancy BMI | 51 | 1.22 | 1.06 | 1.42 | 0.007 |
|  | 1-h glucose | 51 | 1.68 | 1.02 | 2.79 | 0.044 |

*HOMA-IR: Homeostatic Model Assessment for Insulin Resistance, HOMA-B: Homeostasis Model Assessment of β-cell function/insulin secretion, ISSI-2: insulin sensitivity-adjusted secretion index or the insulin disposition index, 1-h glucose: glucose 1-h during oGTT*

*HOMA-IR and HOMA-B were stratified according to their median values in the early postpartum (HOMA-IR ≥ and < 1.55, HOMA-B ≥ and < 24.40).*

^a^ *Predictors entered in the multivariate analysis: pre-pregnancy BMI, fasting glucose, 1-h glucose, ISSI-2, IGI, Matsuda index, fat mass, BMI, weight at 6-8 weeks postpartum*

^b^ *Predictors entered in the multivariate analysis: previous history of GDM, pre-pregnancy BMI, first pregnancy, glucose lowering treatment in pregnancy, BMI, fat mass, weight, ISSI-2, Matsuda index, 1-h glucose, 2-h glucose, HbA1c at 6-8 weeks postpartum*

^c^ *Predictors entered in the multivariate analysis: pre-pregnancy BMI, weight, 1-h glucose, 2-h glucose, fat mass, ISSI-2, Matsuda index at 6-8 weeks postpartum*

^d^ *Predictors entered in the multivariate analysis: previous history of GDM, first pregnancy, glucose lowering treatment in pregnancy, pre-pregnancy BMI, HbA1c, weight, BMI, fat mass, diastolic blood pressure, fasting glucose, Matsuda index,1-h glucose, 2-h glucose, ISSI-2 at 6-8 weeks postpartum*

**Table 4.A** Predictors during pregnancy of glucose intolerance at 1-year postpartum for all GDM women and according to clusters and BMI-categories (model without history of GDM which is only relevant for P≥2).

|  | **Predictors** | **N** | **OR** | **95% C.I.** | | **P value** |
| --- | --- | --- | --- | --- | --- | --- |
|  |  |  |  | **Lower** | **Upper** |  |
| **All women ^a^** | Fasting glucose | 135 | 5.89 | 2.25 | 15.41 | <0.001 |
|  | Gravida | 135 | 0.32 | 0.14 | 0.73 | 0.007 |
|  | Diastolic blood pressure | 135 | 1.07 | 1.02 | 1.13 | 0.004 |
| **Insulin-deficient cluster ^b^** | Fasting glucose | 99 | 6.34 | 1.83 | 22.00 | 0.004 |
| **Insulin-resistant cluster ^c^** | HOMA-IR | 55 | 1.92 | 1.17 | 3.15 | 0.01 |
| **NW* ^d^** | Fasting glucose | 79 | 14.46 | 3.05 | 68.67 | 0.001 |
| **OW/OB* ^e^** | HOMA-IR | 75 | 1.37 | 1.05 | 1.78 | 0.019 |

*^*^ NW: Women with pre-pregnancy normal weight, OW/OB: Women with pre-pregnancy overweight/obesity*

*HOMA-IR: Homeostatic Model Assessment for Insulin Resistance*

*^a^ Predictors entered in the multivariate analysis: first pregnancy, pre-pregnancy BMI, BMI, weight, fasting glucose, HbA1c, fasting insulin, HOMA-IR, fat mass, diastolic blood pressure, systolic blood pressure, glucose lowering treatment in pregnancy*

*^b^ Predictors entered in the multivariate analysis: first pregnancy, pre-pregnancy BMI, weight, fasting glucose in pregnancy*

*^c^ Predictors entered in the multivariate analysis: HbA1c, fasting insulin, HOMA-IR in pregnancy*

*^d^ Predictors entered in the multivariate analysis: pre-pregnancy BMI, HOMA-IR, fasting glucose in pregnancy*

*^e^ Predictors entered in the multivariate analysis: HbA1c, pre-pregnancy BMI, HOMA-IR, fasting insulin in pregnancy*

**Table 4.B** Predictors at 6-8 weeks postpartum of glucose intolerance at 1-year postpartum for all GDM women and according to clusters and BMI-categories (model without the more complex measure ISSI-2).

|  | **Predictors** | **N** | **OR** | **95% C.I.** | | **P value** |
| --- | --- | --- | --- | --- | --- | --- |
|  |  |  |  | **Lower** | **Upper** |  |
| **All women ^a^** | 1-h glucose | 99 | 1.51 | 1.14 | 2.0 | 0.004 |
|  | Pre-pregnancy BMI | 99 | 1.18 | 1.07 | 1.31 | 0.001 |
| **Insulin-deficient cluster ^b^** | 2-h glucose | 103 | 1.62 | 1.14 | 2.31 | 0.007 |
|  | Weight | 103 | 1.06 | 1.02 | 1.11 | 0.006 |
| **Insulin- resistant cluster ^c^** | HbA1c | 39 | 6.56 | 1.09 | 39.32 | 0.04 |
| **NW* ^d^** | 1-h glucose | 59 | 1.77 | 1.16 | 2.7 | 0.008 |
| **OW/OB* ^e^** | 1-h glucose | 37 | 1.85 | 1.035 | 3.30 | 0.038 |
|  | Fat mass | 37 | 1.55 | 1.10 | 2.18 | 0.013 |

**NW: Women with normal weight at 6-8 weeks postpartum, *OW/OB: Women with overweight/obesity at 6-8 weeks postpartum*

*HbA1c:* *Glycated hemoglobin, 1-h glucose: glucose 1-h during oGTT, 2-h glucose: glucose 2-h during oGTT*

*^a^ Predictors entered in the multivariate analysis: previous history of GDM, first pregnancy, glucose lowering treatment in pregnancy,* *pre-pregnancy BMI, weight, triglycerides, BMI, fat mass, systolic blood pressure, diastolic blood pressure, fasting glucose, 1-h glucose, 2-h glucose, fasting insulin, HOMA-IR, Matsuda index., HbA1c, IGI, HOMA-B at 6-8 weeks postpartum*

*^b^ Predictors entered in the multivariate analysis: pre-pregnancy BMI, glucose lowering treatment in pregnancy, weight, BMI, systolic blood pressure, 1-h glucose, 2-h glucose at 6-8 weeks postpartum*

*^c^ Predictors entered in the multivariate analysis: first pregnancy, HbA1c at 6-8 weeks postpartum*

*^d^ Predictors entered in the multivariate analysis: 1-h glucose, 2-h glucose at 6-8 weeks postpartum*

*^e^ Predictors entered in the multivariate analysis: previous history of GDM, pre-pregnancy BMI, gestational weight gain, weight, BMI, fat mass, systolic blood pressure, diastolic blood pressure, fasting glucose, Matsuda index, 1-h glucose, 2-h glucose at 6-8 weeks postpartum*

**Supplementary Figures:**

**Fig. 1 a-f** Clusters characteristic in pregnancy (a-c) and at 6-8 weeks postpartum (d-f).


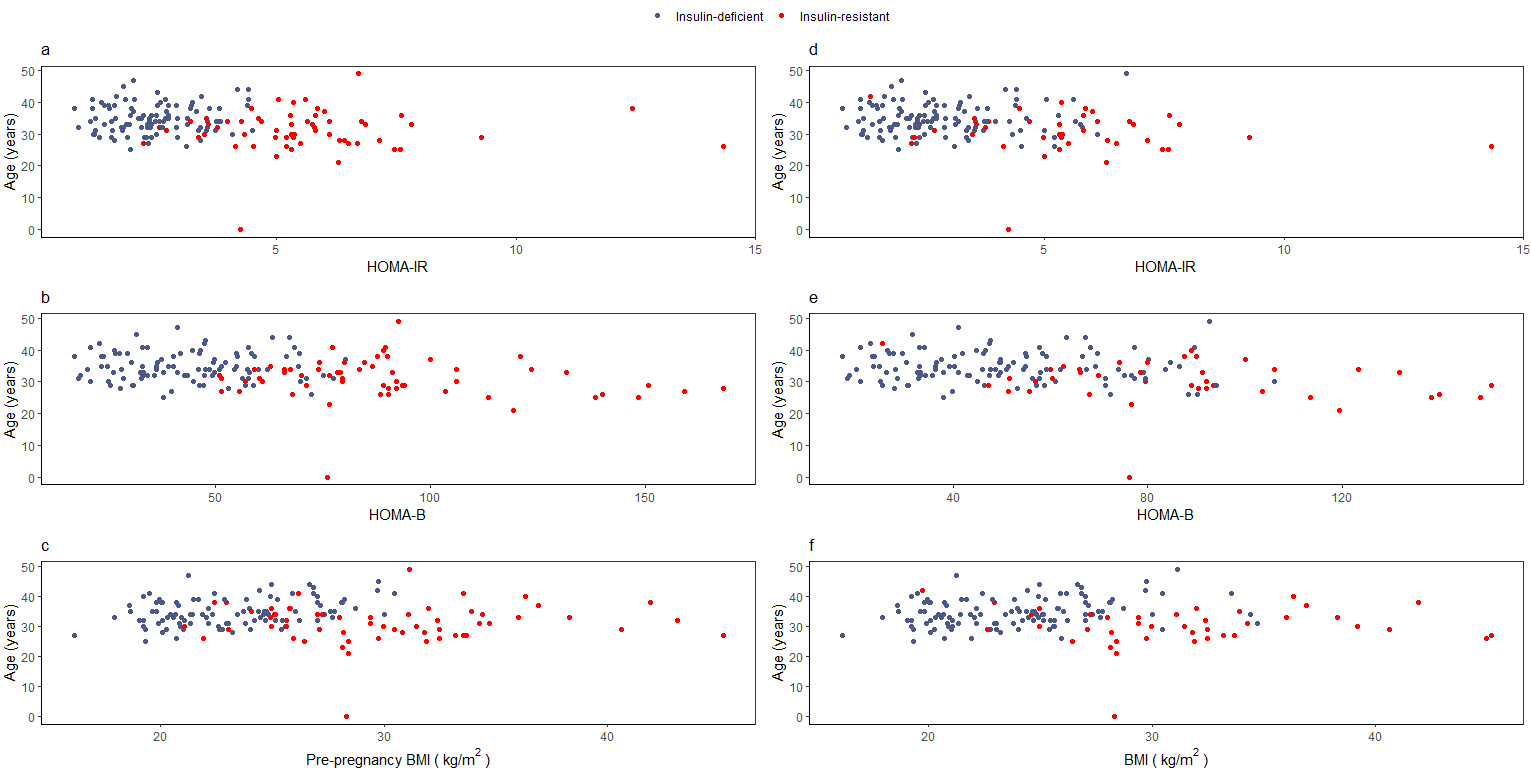


Legend:

These figures show the additional cluster characteristics of the insulin-deficient and insulin-resistant cluster in pregnancy and at 6-8 weeks postpartum.

a) pre-pregnancy age and HOMA-IR in pregnancy b) pre-pregnancy age and HOMA-B in pregnancy, c) pre-pregnancy age and pre-pregnancy BMI, d) pre-pregnancy age and HOMA-IR at 6-8 weeks postpartum, e) pre-pregnancy age and HOMA-B at 6-8 weeks postpartum, f) pre-pregnancy age and BMI at 6-8 weeks postpartum

*BMI= Body Mass Index, HOMA-B=homoeostatic model assessment of β-cell function (insulin secretion), HOMA-IR=homoeostatic model assessment of insulin resistance.*

**Fig. 2** The flow sheet and reasons for drop-out of study participants

Lost to follow-up (n=3)

♦Lost contact (n=3)

Allocated to control arm (n=106)

## Allocation

Randomized (n=211)

Excluded (n=750)

♦  Not meeting inclusion criteria (n=432)

♦  Declined to participate (n=294)

♦  Other reasons (n=24)

Assessed for eligibility (n=961)

Allocated to intervention arm (n=105)

## 6-8 pp weeks follow-up

Lost to follow-up (n=7)

♦Lost contact (n=5)

♦Diagnosis revised after allocation (n=2)

## 1-year pp follow-up

Lost to follow-up (n=8)

♦Lost contact (n=6)

♦New pregnancy (n=2)

Discontinued intervention (give reasons) (n= )

Lost to follow-up (n=14)

♦Lost contact (n=11)

♦New pregnancy (n=3)

## Analysis

Data at 1-year postpartum (n=84)

Data at 1-year postpartum (n=95)

**Legend:** *pp=postpartum*
